# Real-world efficacy of intravitreal faricimab for neovascular age-related macular degeneration: a systematic review

**DOI:** 10.1186/s40942-024-00566-0

**Published:** 2024-07-12

**Authors:** Nasratullah Nasimi, Safiullah Nasimi, Jakob Grauslund, Anna Stage Vergmann, Yousif Subhi

**Affiliations:** 1 Department of Ophthalmology, Odense University Hospital, Odense, Denmark; 2 Department of Clinical Research, University of Southern Denmark, Campusvej 55, 5230 Odense, Denmark; 3 Steno Diabetes Center Odense, Odense University Hospital, Odense, Denmark; 4 Department of Ophthalmology, Vestfold Hospital Trust, Tønsberg, Norway; 5 Department of Ophthalmology, Rigshospitalet, Copenhagen, Denmark; 6 Department of Ophthalmology, Zealand University Hospital, Roskilde, Denmark

**Keywords:** Age-related macular degeneration, Faricimab, Efficacy, Real-world evidence, Systematic review

## Abstract

**Background:**

To systematically review the real-world outcomes of intravitreal faricimab treatment in patients with neovascular age-related macular degeneration (nAMD) to evaluate its efficacy and safety in clinical settings. This study was conducted due to the need for real-world evidence to complement the findings from controlled clinical phase-III trials.

**Methods:**

A systematic literature search was conducted on March 17, 2024, across 11 databases, utilizing search terms specifically tailored each database. All studies were reviewed qualitatively with specific focus on the outcomes of interest: the best-corrected visual acuity (BCVA), the central retina thickness (CRT), and the burden of therapy.

**Results:**

We identified a total of 22 eligible studies of 1762 eyes from 1618 patients with nAMD. Studies reported that intravitreal faricimab injections maintained BCVA in patients with previously treated eyes and demonstrated statistically significant improvement in patients with treatment-naïve eyes. The CRT was reduced after intravitreal faricimab therapy. Faricimab was well-tolerated, with no significant safety concerns identified, and reduced the overall burden of therapy.

**Conclusion:**

Real-world studies corroborate the conclusions drawn from phase-III trials regarding faricimab treatment, demonstrating improvement in both visual and anatomical outcomes. Additionally, no significant safety issues were identified, as the treatment was generally well-tolerated and reduced the overall burden of therapy in the real-world settings.

**Supplementary Information:**

The online version contains supplementary material available at 10.1186/s40942-024-00566-0.

## Introduction

Neovascular age-related macular degeneration (nAMD) is the leading cause of irreversible vision loss among the elderly in developed world [1–3]. The disease pathophysiology involves the secretion of vascular endothelial growth factor (VEGF), which lead to the formation of fragile blood vessels that ultimately result in visual impairment [4]. Intravitreal injections of anti-VEGF agents have been effective in improving the functional and anatomical properties of eyes with nAMD [5, 6]. While anti-VEGF agents are effective, their limitations include the requirement for frequent injections and need for long-term treatment for nAMD [7]. To overcome these boundaries, the attention has led to finding more sustainable treatment solutions, including longer acting drugs or agents targeting other pathways [8, 9].

Intravitreal faricimab (Vabysmo, F. Hoffmann-La Roche AG, Basel, Switzerland) is a novel anti-angiopoietin-2 (Ang-2) and anti-VEGF bispecific agent approved by Food and Drug Administration (FDA) and European Medicines Agency (EMA) for treatment of nAMD and diabetic macular edema (DME). Ang-2 functions as a proinflammatory cytokine, which promote neovascularization in the aged retinal micromilleu, and enhances the effect of VEGF on neovascularization [4, 10, 11]. The approval of faricimab was based on four phase 3 studies. TENAYA and LUCERNE for nAMD [12] and YOSEMITE and RHINE for DME [13]. All studies reported visual and anatomical benefits. The mean best-corrected visual acuity (BCVA) change from baseline with faricimab was non-inferior to aflibercept in both TENAYA (5.8 vs. 5.1 ETDRS letter) and LUCERNE (6.6 vs. 6.6 ETDRS letters) [12]. Rates of ocular adverse events were comparable between faricimab and aflibercept [12].

However, the results of clinical trials may not necessarily reflect the results when applied in real-world context [14, 15]. Patients in real-world clinics may not always fit the eligibility criteria of clinical trials and circumstances around routine clinic may differ from those in controlled trials. Furthermore, real-world studies can give insight into outcomes from switching therapies, *e.g.*, in this case from other intravitreal anti-VEGF therapies to faricimab. Therefore, this study aims to evaluate the efficacy and durability of action of intravitreal faricimab in real-world studies of patients with nAMD.

## Methods

### Protocol and registration

We followed the recommendations of the Cochrane Handbook for the design and conduct of our study [16]. Our protocol registered at PROSPERO (protocol no. CRD42024537080). We followed the Preferred Reporting Items for Systematic Reviews and Meta-Analysis (PRISMA) [17]. According to Danish law, no institutional review board approval is not relevant for systematic reviews.

### Eligibility criteria

*Population* Studies of patients with neovascular AMD. We did not restrict patient population based on any previous treatment. We only considered studies of human patients.

*Exposure* Intravitreal injection therapy using faricimab 6 mg (0.05 mL).

*Outcomes* Change from baseline to follow-up in CRT and BCVA as well as the burden of therapy (*i.e.*, number of injections/therapies needed).

*Study design* Any prospective or retrospective studies with original data of real-world evidence. Case reports, non-peer-reviewed publications and conference abstracts were not eligible. We only considered studies disseminated in English for practical purposes. No restriction was made on the geographical origin of the study or the date of study publication.

### Information sources, literature search, and study selection

One trained author (Y.S.) conducted a systematic literature search in 11 databases *(PubMed, Embase, Web of Science Core Collection, BIOSIS Previews, Current Contents Connect, Data Citation Index, Derwent Innovations Index, KCI-Korean Journal Database, ProQuest Dissertations & Theses Citation Index, SciELO Citation Index, and the Cochrane Library).* All searches were conducted on 17 March 2024. Literature search details for individual databases are available in Supplementary file 1.

One author (Y.S.) removed all duplicates and obviously irrelevant reports. Two authors (N.N. and S.N.) independently screened full text of the remaining records for eligible studies. Reference lists were screened for further eligible studies. Disagreements between authors were discussed until consensus, and if consensus could not be reached, a third author (Y.S.) made the final decision.

### Data collection and extraction, risk of bias within studies, and data synthesis

Two authors (N.N. and S.N.) independently extracted data and evaluated risk of bias within studies. Data were extracted on study and population characteristics, treatment details, and clinical outcomes at baseline and follow-up. Since we expected studies to be primarily retrospective cohort studies, we used the Newcastle–Ottawa Scale for the evaluation of risk of bias within studies [18]. Disagreements between authors were discussed until consensus, and if consensus could not be reached, a third author (Y.S.) made the final decision.

All studies were reviewed qualitatively in text and in tables. Due to the heterogeneity of the available studies, meaningful quantitative analyses were not possible.

## Results

### Study selection process

**Fig. 1:**
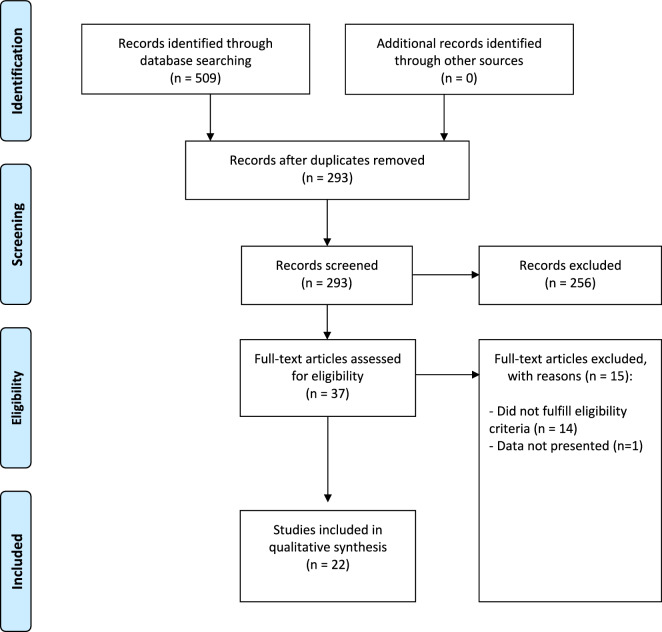
PRISMA flow diagram of study selection process

Our literature search identified 509 records of which 216 were duplicates and 256 were obviously irrelevant. The 37 remaining records were examined in full text for eligibility. Of these, 15 were excluded as they did not fulfill our eligibility criteria. Thus 22 studies were eligible for inclusion in our review (Fig. 1).

### Characteristics of studies

We identified 22 eligible studies of real-world evidence published between March 23, 2023, and February 28, 2024 [19–40]. These studies summarized data from 1762 eyes of 1618 patients. Studies reported outcomes from patients in USA (n = 883), Japan (n = 532), UK (n = 131), Switzerland (n = 26), and Denmark (n = 46). Patients had a mean age of 70.2–83.0 years and 776 (52%) were females. Study designs were predominantly retrospective cohorts, although three studies were retrospective interventional studies. Seventeen studies were studies of switchover from aflibercept, bevacizumab, ranibizumab, or brolucizumab to faricimab. Study and population characteristics are summarized in detail in Table 1.

**Table 1:** Study characteristics

Reference	Design	Country	Mean age (years)	Biological sex	Patients, N	Treatment naïve eyes, N	Previously treated eyes, N	Mean follow-up duration
Cheng et al. [19]	Interventional study	USA	81.8	Male: 4 Female: 7	11	0	12	3.4 months
Grimaldi et al. [20]	Prospective cohort study	Switzerland	82.0	Male: 11 Female: 14	26	0	26	30.2 weeks
Hara et al. [21]	Retrospective cohort study	Japan	79.4	Male: 18 Female: 11	29	30	0	3.0 months
Hikichi [22]	Retrospective observational study	Japan	81.1	Male: 28 Female: 20	48	0	48	6.0 months
Inoda et al. [23]	Retrospective cohort study	Japan	76.7	Male: 49 Female: 26	75	0	80	Not specified
Kataoka et al*.* [24]	Retrospective cohort study	Japan	78.3	Male: 17 Female: 36	53	0	53	6.0 months
Khanani et al*.* [25]	Retrospective cohort study	USA	79.8	Male: 150 Female: 185	335	39	337	6.0 months
Kishi et al*.* [26]	Retrospective cohort study	Japan	80.1	Male: 34 Female: 21	55	0	55	16.1 weeks
Leung et al*.* [27]	Retrospective cohort study	USA	80.1	Male: 109 Female: 77	186	0	190	35 weeks
Maruyama-Inoue et al*.* [28]	Retrospective cohort study	Japan	75.4	Male: 27 Female: 19	46	47	0	4 months
Matsubara et al*.* [29]	Retrospective cohort study	Japan	78.5	Male: 11 Female: 3	14	1	13	4 weeks
Matsumoto et al*.* [30]	Retrospective interventional study	Japan	80.3	Male: 15 Female: 14	29	30	0	12 months
Mukai et al*.* [31]	Retrospective cohort study	Japan	76	Male: 41 Female: 20	61	63	0	3 months
Ng et al*.* [32]	Retrospective cohort study	UK	79.2	Male: 21 Female: 33	54	0	63	7 months
Pandit et al*.* [33]	Retrospective interventional study	USA	79.9	Male: 92 Female: 99	191	0	218	160 days
Raimondi et al*.* [34]	Retrospective observational study	UK	79.1	Male: 45 Female: 43	68	0	81	Not specified
Rush [35]	Retrospective cohort study	USA	75.7	Male: 28 Female: 26	54	0	54	12 months
Schneider et al*.* [36]	Retrospective cohort study	Denmark	76.4	Male: 25 Female: 21	46	0	50	4 weeks
Stanga et al*.* [37]	Retrospective cohort study	UK	76.4	Male: 4 Female: 5	9	3	8	12.5 weeks
Szigiato et al*.* [38]	Retrospective cohort study	USA	80.7	Male: 32 Female: 74	106	0	126	24.3 weeks
Tamiya et al*.* [39]	Prospective observational study	Japan	77.2	Male: 19 Female: 6	25	0	25	4 months
Tanaka et al*.* [40]	Prospective observational study	Japan	75.4	Male: 13 Female: 10	23	23	0	4 months

*UK* United Kingdom, *USA* United States of America

Studies evaluated treatment of 6.0 mg faricimab. Seven studies did not specify the dose of faricimab but were not excluded, as their inclusion remained pertinent to assessing the safety profile of faricimab. Seventeen studies were switch-over from a previous treatment to faricimab, and five studies was on treatment-naïve eyes. The follow-up regimen ranged from three to 12 months. Treatment details and follow-up regimens for each study are summarized in Table 2.

**Table 2:** Treatment details in individual studies

Reference	Treatment	Follow-up regimen
Cheng et al. [19]	Patient who showed inadequate response to prior treatments were switched to monthly injections of faricimab. Faricimab dose was 6.0 mg for each intravitreal injection	Mean duration of 3.4 months after starting faricimab treatment
Grimaldi et al. [20]	Patients received a loading dose of four 4-weekly 6.0 mg injections of intravitreal faricimab, followed by an eight-week extension. A treat-and-extend regimen was then adopted	Treatment intervals were adjusted depending on disease activity and could be maintained, extended, or shortened
Hara et al. [21]	Patients received 3 monthly 6.0 mg injections of intravitreal faricimab. Assessment was made at baseline and at one-, two-, and three- months post treatment initiations	Follow-up visits were scheduled at one-, two-, and three-months after the initial treatment
Hikichi [22]	Patients switched to faricimab and followed on a treat-and-extend regimen. Maintaining the same interval as their previous treatment. The interval was adjusted by 2 weeks based on OCT-assessed disease activity with a minimum 4 weeks and maximum of 16-weeks. Each intravitreal injection of faricimab was 6.0 mg	Structured to monitor the efficacy and safety of faricimab over 6-months period following the switch
Inoda et al. [23]	Patient previously treated with aflibercept or brolucizumab received a single initial 6.0 mg intravitreal faricimab injection. Subsequent treatments were scheduled based on their pre-existing treatment intervals, without a loading phase	After the initial faricimab injection, treatment intervals were maintained as before. Patients continued their regular visit intervals (± 1 week)
Kataoka et al*.* [24]	Patients receiving monthly aflibercept were switched to monthly faricimab until no SRF, IRF or sub-RPE fluid was detected on OCT scans, or up to four injections. Afterwards, treatment intervals were extended by two- to four-week adjustment up to 8 weeks. The dose of faricimab was not specified	After the switch to faricimab, patients were monitored monthly during to assess SRF, IRF, and sub-RPE changes using OCT-scan
Khanani et al*.* [25]	Patients received monthly injections for the first 3 months, as the loading phase. Afterward, treatment intervals were extended based on response. The dose of faricimab was not specified	Patients were closely monitored with follow-up visits after the first and third injections to assess outcomes
Kishi et al*.* [26]	Patient received three consecutive faricimab injections without a loading phase. Following the treat-and-extend regimen, intervals were based on the pre-switch schedule and adjusted by 2 weeks according to OCT findings. The minimum interval was set at 4 weeks. The dose of faricimab was not specified	Patients had ophthalmologic examinations and OCT scans before each of the three injections and at regular intervals afterward to assess the effectiveness of faricimab
Leung et al*.* [27]	The decision to switch to faricimab was made by the treating physician, often due to persistent fluid or inability to extend intervals beyond four to 6 weeks. Patient received monthly faricimab doses for the first three injections. Afterward, dosing intervals could be extended by 2 weeks based on clinical assessments. Each faricimab dose was 6.0 mg	Patients were followed for approximately 35 weeks after the initial faricimab treatment. Assessment occurred after each injection with an evaluation at 3 months post-initiation of faricimab
Maruyama-Inoue et al*.* [28]	This study evaluated two different intravitreal anti-VEGF agents, brolucizumab and faricimab. They were administered for two different groups and time. Each patient received three consecutive monthly injections. The dose of faricimab was not specified	Follow-up visits were scheduled for one-, two-, and 4 months post initial treatment to monitor and compare the efficacy and safety of the treatments
Matsubara et al*.* [29]	Three anti-VEGF agents were used: aflibercept, brolucizumab and faricimab. Each patient received an initial intravitreal injection of one of these drugs. The dose of faricimab was not specified	The anterior chamber was evaluated by AFV and retinal function by flicker ERG before the injection, two- and four-weeks post-treatment
Matsumoto et al*.* [30]	Patients received 3 monthly intravitreal injection of faricimab as loading phase. Following the loading phase, patients entered a maintenance phase with treat-and-extend regimen, adjusting intervals based on individual responses. The dose of faricimab was not specified	After the initial loading phase, the injection intervals were extended by 4 weeks if the macula was dry or shortened by 4 weeks if it was not. The treatment intervals ranged from eight to 16 weeks
Mukai et al*.* [31]	Patients received three consecutive monthly intravitreal injections of faricimab as loading therapy. Faricimab dose was 6.0 mg for each intravitreal injection	After the initial loading phase, patients were closely monitored for 3 months. The primary evaluation points were at one-, two- and three-months post-treatment initiation
Ng et al*.* [32]	Patients switched to faricimab in three ways. Initially, they received loading doses, followed by treat-and-extend protocol If SD-OCT showed improvement, intervals were extended by 4 weeks. Without improvement or if worsening occurred, treatments continued with 4 monthly doses until the next review. Each faricimab dose was 6.0 mg	After the loading phase, patients were assessed starting at 4 weeks post-treatment. If improvement were observed, the injection intervals increased by two or 4 weeks, up to 16 weeks. SD-OCT-imaging was performed before each injection and reviewed to decide treatment intervals
Pandit et al*.* [33]	Patients received ≥ four intravitreal injections of faricimab after the switch from a previous treatment. Faricimab dose was 6.0 mg for each intravitreal injection	Patient underwent regular follow-up visits: Before the first injection, at the following three injections, and a final visit after the last injection
Raimondi et al*.* [34]	Patients switched from intravitreal aflibercept to faricimab after receiving at least six consecutive aflibercept injections. The switch involved two or three initial four-weekly loading doses of faricimab, based on clinician preference, with assessments 4 weeks after the injections. Each faricimab dose was 6.0 mg	Depending on the regimen, patients received two or three loading doses of faricimab at four-week intervals. Post-loading phase, treatment intervals were adjusted based on IRF, SRF, and CMT
Rush [35]	Patients switched from aflibercept to faricimab and received monthly faricimab injections for the first 3 months. After this loading phase, OCT scans assessed retinal edema. If edema was eliminated, treatment intervals were extended by one-two weeks. The dose of faricimab was not specified	Patients underwent regular follow-up visits with OCT to monitor central macular thickness and fluid presence. Follow-up lasted 12 months to assess long-term efficacy and safety
Schneider et al*.* [36]	Patients received intravitreal injections of faricimab, administered by trained medical personnel following local clinical standard protocol. Faricimab dose was 6.0 mg for each intravitreal injection	Patients had follow-up examinations 4 weeks after the faricimab injection. If the macula was dry, weekly check-ups were scheduled to monitor treatment response durability
Stanga et al*.* [37]	The treatment consisted of an intravitreal injection of faricimab at a dosage of 6.0 mg, using a standardized protocol that assessed treatment efficacy and safety at multiple follow-up intervals	After the initial faricimab injection, patients were monitored for several months with primary follow-up at 1 months and subsequently visit based on the individual treatment response
Szigiato et al*.* [38]	Patients received ≥ three intravitreal injections of faricimab after the switch from a previous treatment. Faricimab dose was 6.0 mg for each intravitreal injection	Patients underwent follow-up visits after each injection where the outcomes were assessed. The visit was at the baseline and after each injection
Tamiya et al*.* [39]	Patients received a single dose of 6.0 mg Faricimab, and patients were observed without additional doses for up 2 months	Clinical data were collected at baseline and 2 months after the faricimab injection. Visual acuity and SD-OCT were performed at 4 months, and results from baseline and 4 months were analyzed
Tanaka et al*.* [40]	Three monthly intravitreal injections of Faricimab at a dosage of 6.0 mg	The follow-up evaluations were conducted 2 months after the third injection, referred as month four in the study. If the patient didn’t show SRF or IRF at this point, further clinical data were collected at months five

*AVF* aqueous flare value, *CMT* central macular thickness, *ERG* electroretinography, *IRF* intraretinal fluid, *OCT-scan* optical coherence tomography scan, *SD-OCT* spectral domain optical coherence tomography, *SRF* sub-retinal fluid; sub-*RPE* sub-retinal pigment epithelium

### Efficacy of faricimab in treatment naïve eyes with nAMD

Five studies investigated the effect of faricimab in treatment-naïve eyes with nAMD [21, 28, 30, 31, 40]. Hara et al*.* [21] reported no significant improvement in BCVA (0.46 ± 0.41 logMAR before the treatment vs. 0.44 ± 0.45 logMAR after), CRT decreased from 498 ± 227 μm to 217 ± 74 μm, and no adverse events were reported [21]. Maruyama-Inoue et al*.* [28] reported that BCVA improved (0.36 ± 0.33 logMAR before the treatment vs. 0.28 ± 0.32 logMAR after), that central foveal thickness (CFT) decreased from 407 ± 187 μm to 226 ± 94 μm, and that no adverse event was reported [28]. Matsumoto et al*.* [30] reported that BCVA improved (0.32 ± 0.40 logMAR before treatment vs. 0.17 ± 0.33 logMAR after) and that one eye of 30 eyes in study in total developed intraocular inflammation (IOI) [30]. The mean intended injection interval at the last visit was 12.7 weeks [30]. Mukai et al*.* [31] reported that BCVA improved (0.40 ± 0.42 logMAR before treatment vs. 0.32 ± 0.43 logMAR after), that CRT decreased from 357 ± 165 μm to 175 ± 91 μm, and two cases of retinal pigment epithelium (RPE) tears [31]. After 3 months, 82% of eyes were reported to have obtained a dry macula [31]. Tanaka et al*.* [40] reported that BCVA improved (0.29 ± 0.30 logMAR before treatment vs. 0.18 ± 0.32 logMAR after the treatment, p = 0.00049), that CRT decreased from 325 ± 193 μm to 164 ± 90 μm, and one case of RPE tear [40]. After 4 months, 77% of eyes were reported to have obtained a dry macula [40].

### Switchover to faricimab treatment in eyes with nAMD previously treated with other anti-VEGF therapies

Fourteen studies evaluated the effect a switchover from any previous intravitreal anti-VEGF treatment to faricimab [19, 20, 22–24, 26, 27, 32–36, 38, 39]. Cheng et al*.* [19] reported that BCVA remained stable (0.59 ± 0.45 logMAR before vs. 0.58 ± 0.45 logMAR after the switch), that median central subfield thickness (CST) decreased from 342 μm to 318 μm, and no serious adverse events [19]. Grimaldi et al*.* [20] reported that BCVA remained stable (median 0.35 logMAR before vs. 0.30 logMAR after the switch), that median CST decreased from 357 μm to 292 μm, one case of RPE tear, and no serious adverse events [20]. Time interval between the injections increased from 4.0 weeks to 6.0 weeks after the switch [20]. Hikichi [22] reported that BCVA remained stable (mean 0.38 logMAR before vs. 0.31 logMAR after the switch), that mean CFT decreased from 372 μm to 272 μm, and no adverse events [22]. The mean interval of injections increased from 6.7 weeks to 10.5 weeks after the switch [22]. Inoda et al*.* [23] reported that BCVA remained stable (0.34 ± 0.37 logMAR before vs. 0.36 ± 0.40 logMAR after the switch), that mean CST remained stable (242 ± 72 μm before the switch vs. 242 ± 82 μm after), and no adverse events [23]. The treatment intervals were similar to those before the switch [23]. Kataoka et al*.* [24] reported that BCVA remained stable (0.3 ± 0.4 logMAR before the switch vs. 0.3 ± 0.4 logMAR after), that mean CRT decreased from 320 ± 181 μm to 302 ± 143 µm, and reported one case of mild iritis [24]. The mean interval of injections increased from 4.4 weeks to 8.7 weeks after the switch [24]. Kishi et al*.* [26] reported that BCVA remained stable (0.26 ± 0.34 logMAR before the switch vs. 0.23 ± 0.37 logMAR after), that mean CRT significantly decreased from 320 ± 179 μm to 312 ± 189 μm, and one case of RPE tear [26]. The mean interval of injections increased from 5.9 weeks to 6.1 weeks after the switch [26]. Leung et al*.* [27] reported that BCVA improved (0.33 ± 0.32 logMAR before the switch vs. 0.27 ± 0.32 logMAR after) and that CRT decreased from 312 ± 87 μm to 287 ± 71 μm. Two eyes developed endophthalmitis, four eyes developed RPE tears, and three eyes developed subretinal hemorrhages [27]. The interval of injections increased from 5.2 weeks to 7.6 after the switch [27]. Ng et al*.* [32] reported that BCVA remained stable (0.47 ± 0.34 logMAR before the switch vs. 0.49 ± 0.36 after) and that central macular thickness (CMT) decreased from 344 ± 96 μm to 320 ± 98 μm [32]. Pandit et al*.* [33] reported that BCVA remained stable (0.58 ± 0.54 logMAR before the switch vs. 0.55 ± 0.52 logMAR after), that mean CFT decreased from 355 μm to 306 μm, and no adverse events [33]. The interval of injections was increased from 36 to 43 days [33]. Raimondi et al*.* [34] reported that BCVA remained stable (65 ± 12 ETDRS letters before the switch vs. 65 ± 13 ETDRS letters after), that CMT decreased from 330 ± 103 μm to 287 ± 73 μm, and no adverse events [34]. Rush (2023) reported that BCVA improved (mean 0.72 logMAR before the switch to 0.59 logMAR), that mean CMT decreased from 395 µm to 350 μm, and no adverse events [35]. Dry macula with a treatment interval ≥ 8 weeks was achieved in 31.5% (17/54) [35]. Schneider et al*.* [36] reported that BCVA remained stable (median 74 ETDRS letters before the switch vs. 74 after), that median CRT decreased from 252 μm to 232 μm, and no adverse events [36]. Szigiato et al*.* [38] reported that BCVA remained stable (median 62.9 ETDRS letters before the switch vs. 62.7 ETDRS letters after) and that CRT decreased from 267 ± 65 μm to 250 ± 59 μm [38]. One patient developed IOI requiring cessation of further intravitreal faricimab injections [38]. No other adverse event was reported [38]. The interval of injections increased from 5.6 weeks to 6.8 weeks [38]. Tamiya et al*.* reported that BCVA remained stable (0.21 ± 0.18 logMAR before the switch vs. 0.24 ± 0.13 logMAR after), that CRT decreased from 193 ± 109 μm to 182 ± 105 μm, and no adverse events [39]. Notably, 25% of the eyes that showed dry macula at month two had no fluid recurrence for up to 4 months [39].

### Studies in which the entire nAMD treatment service, *i.e.*, both treatment-naïve and existing patients, are switched over to faricimab

Three studies included patients with both treatment-naïve eyes and those who had previously received treatment [25, 29, 37]. Khanani et al*.* [25] reported that BCVA improved in both the switch-over eyes (from mean 58 ETDRS letters to 61 ETDRS letters) as well as the treatment-naïve eyes (from mean 51 ETDRS letters to 59 ETDRS letters), with the latter group experiencing the greatest improvement [25]. The mean CST decreased significantly in both the switch-over and the treatment-naïve eyes [25]. One case of IOI was reported [25]. No serious adverse events were reported [25]. Matsubara et al*.* [29] reported that BCVA improved (median 0.046 logMAR before treatment vs. 0.072 logMAR after), that median CST decreased from 329 μm to 319 μm, and no adverse events [29]. Stanga et al*.* [37] reported that BCVA improved both in treatment-naïve eyes (from 0.33 ± 0.29 logMAR to 0.30 ± 0.29 logMAR) and switch-over eyes (from 0.61 ± 0.75 logMAR to 0.39 ± 0.54 logMAR) [37]. The CRT decreased both in treatment-naïve eyes (from 875 ± 511 μm to 537 ± 352 μm) and in switch-over eyes (from 256 ± 13 μm to 245 ± 15 μm). No adverse events were reported [37]. A complete resolution of SRF was observed in six out of eight eyes (75%) and of IRF in 2 out of 3 eyes (66.67%) [37].

### Risk of bias within studies

The evaluation of risk of bias within studies was made using the Newcastle–Ottawa Quality Assessment Scale for cohort studies. All studies were evaluated on selection-, comparability-, and outcome categories. All studies scored 0 point in non-exposed cohort (selection #2) as all studies, except two studies, involved a switch-over from a previous treatment to faricimab. The two studies were Hara et al*.* [21] and Maruyama-Inoue et al. [28], which investigated the relationship between a previous treatment and faricimab. All studies received a high-quality score, and Hara et al*.* [21] and Maruyama-Inoue et al*.* [28] scored a maximum score. Details of the risk of bias within studies are summarized in Table 3.

**Table 3:** Risk of bias within individual studies

Reference	Selection				Comparability	Outcome			Quality score
	#1	#2	#3	#4	#1	#1	#2	#3	
Cheng et al. [19]	✯	–	✯	✯	✯	✯	✯	✯	7
Grimaldi et al. [20]	✯	–	✯	✯	✯	✯	✯	✯	7
Hara et al. [21]	✯	✯	✯	✯	✯	✯	✯	✯	8
Hikichi [22]	✯	–	✯	✯	✯	✯	✯	✯	7
Inoda et al. [23]	✯	–	✯	✯	✯	✯	✯	✯	7
Kataoka et al*.* [24]	✯	–	✯	✯	✯	✯	✯	✯	7
Khanani et al*.* [25]	✯	–	✯	✯	✯	✯	✯	✯	7
Kishi et al*.* [26]	✯	–	✯	✯	✯	✯	✯	✯	7
Leung et al*.* [27]	✯	–	✯	✯	✯	✯	✯	✯	7
Maruyama-Inoue et al*.* [28]	✯	✯	✯	✯	✯	✯	✯	✯	8
Matsubara et al*.* [29]	✯	–	✯	✯	✯	✯	✯	✯	7
Matsumoto et al*.* [30]	✯	–	✯	✯	✯	✯	✯	✯	7
Mukai et al*.* [31]	✯	–	✯	✯	✯	✯	✯	✯	7
Ng et al*.* [32]	✯	–	✯	✯	✯	✯	✯	✯	7
Pandit et al*.* [33]	✯	–	✯	✯	✯	✯	✯	✯	7
Raimondi et al*.* [34]	✯	–	✯	✯	✯	✯	✯	✯	7
Rush [35]	✯	–	✯	✯	✯	✯	✯	✯	7
Schneider et al*.* [36]	✯	–	✯	✯	✯	✯	✯	✯	7
Stanga et al*.* [37]	✯	–	✯	✯	✯	✯	✯	✯	7
Szigiato et al*.* [38]	✯	–	✯	✯	✯	✯	✯	✯	7
Tamiya et al*.* [39]	✯	–	✯	✯	✯	✯	✯	✯	7
Tanaka et al*.* [40]	✯	–	✯	✯	✯	✯	✯	✯	7

The Newcastle–Ottawa Quality Assessment Scale for Cohort Studies evaluates categories within three domains: Selection, Comparability, and Outcome. Categories within Selection are (#1) representativeness of the exposed cohort, (#2) selection of the non-exposed cohort, (#3) ascertainment of exposure, and (#4) demonstration that outcome of interest was not present at start of study. For Comparability, only one category is evaluated in (#1) comparability of cohorts based on the design or analysis. Categories within Outcome are (#1) assessment of outcome, (#2) was follow-up long enough for outcomes to occur, and (#3) adequacy of follow-up of cohorts. The quality score is a summary of number of stars across all categories within each study

## Discussion

In this systematic review, our aim was to evaluate the efficacy and durability in intravitreal faricimab treatment in patients with nAMD. Overall, existing real-world evidence presents a pattern of BCVA improvement and CRT decrease in treatment-naïve eyes, and stable BCVA with longer treatment duration in switch-over eyes. Many patients were able to achieve a dry macula, also in cases of switch-over from other intravitreal anti-VEGF therapies with inadequate treatment response. Overall, studies also reported that faricimab was well-tolerated with only rare incidences of adverse events (retinal pigment epithelium tears, mild iritis, endophthalmitis, subretinal hemorrhages, or IOI).

Preclinical studies of Ang-2 inhibition in choroidal neovascularization in mice showed that inhibiting Ang-2 led to reduced vascular leakage and lesion numbers [11, 41]. Combination therapy with both Ang-2 inhibitor and anti-VEGF was superior to anti-VEGF alone [11, 41]. These findings in preclinical studies underscore the pathophysiological rationale for the efficacy of the bispecific anti-Ang-2 and anti-VEGF faricimab.

Hara et al*.* [21] and Maruyama-Inoue et al*.* [28] compared faricimab with another anti-VEGF treatments in their respective cohorts in a real-world setting. In contrast to the findings of the TENAYA and LUCERNE trials, which concluded that faricimab was non-inferior to aflibercept [12]; Hara et al*.* [21] concluded that faricimab was inferior to aflibercept in terms of BCVA gain [21]. Both the group of faricimab treated eyes and the aflibercept treated eyes seemed to be comparable in their baseline characteristics [21]. More comparative real-world studies, preferably with larger study sample size are warranted to further explore this discrepancy between the real-world evidence as suggested by Hara et al. [21] and the results of the TENAYA and LUCERNE trials [12]. Maruyama-Inoue et al*.* [28] attributed the rapid improvement in BCVA in intravitreal brolucizumab treatment group to differences in molecular weight and affinity for VEGF between the two anti-VEGF treatments [28]. Brolucizumab has a lower molecular weight, which might facilitate the delivery of more active molecules per injection and potentially allow for more effective tissue penetration and increased efficacy [42]. However, one complicating factor of brolucizumab therapy is that it is associated with a different safety profile in terms of a higher incidence of IOI, retinal vasculitis, and retinal vascular occlusion [43].

Taken together, studies illustrated that a switch to faricimab allowed for a statistically significant extension of treatment intervals, which may reduce injection frequency and present a possibility to reduce the logistical, financial, and emotional burdens associated with regular hospital visits. Thus, real-world evidence as presented in this review suggests that faricimab therapy lowers the burden of treatment for patients with nAMD.

There are several limitations to our systematic review. The included studies lack a control group for treatment comparison, which makes it difficult to draw definitive conclusions when comparing to other anti-VEGF therapies. In addition, most studies were relatively small retrospective studies, which in terms of clinical evidence has certain biases. However, studies were available from different centers from different countries, which is a benefit in terms of the generalizability and applicability of our findings. Moreover, a limitation of this systematic review is that it was not a Cochrane review, which methodologically is seen as gold standard among many colleagues.

In conclusion, the existing real-world evidence of intravitreal faricimab therapy find that it can maintain BCVA in the majority of the patients, reduces the CRT, and does this while reducing the burden of therapy. These real-world studies align with the results from the controlled experimental trials [12]. Therefore, faricimab as a first-line therapy holds potential to, at least to a certain degree, alleviate the important burden of therapy in patients with nAMD [7, 44].

### Supplementary Information

Additional file 1. Details of the literature search across different databases.

## Data Availability

All data generated or analysed during this study are included in this published article and its supplementary information files.

## Abbreviations

Ang-2: Angiopoietin-2
BCVA: Best-corrected visual acuity
CFT: Central foveal thickness
CMT: Central macular thickness
CRT: Central retinal thickness
CST: Central subfield thickness
DME: Diabetic macular edema
EMA: European Medical Agency
FDA: Food and Drug Administration
nAMD: Neovascular age-related macular degeneration
RPE: Retinal pigment epithelium
VEGF: Vascular endothelial growth factor

## References

[1] Li JQ, Welchowski T, Schmid M, Mauschitz MM, Holz FG, Finger RP (2020). Prevalence and incidence of age-related macular degeneration in Europe: a systematic review and meta-analysis. Br J Ophthalmol.

[2] Sedeh FB, Scott DAR, Subhi Y, Sørensen TL (2017). Prevalence of neovascular age-related macular degeneration and geographic atrophy in Denmark. Dan Med J.

[3] van Dijk EHC, Holtz JK, Sirks MJ, Larsson JME, Diederen RMH, Schlingemann RO, Boon CJF, Subhi Y (2022). European prevalence of polypoidal choroidal vasculopathy: a systematic review, meta-analysis, and forecasting study. J Clin Med.

[4] Rozing MP, Durhuus JA, Krogh Nielsen M, Subhi Y, Kirkwood TB, Westendorp RG, Sørensen TL (2020). Age-related macular degeneration: a two-level model hypothesis. Prog Retin Eye Res.

[5] Salehi MA, Frounchi N, Zakavi SS, Mohammadi S, Harandi H, Shojaei S, Gouravani M, Fernando AJ (2024). Retinal and choroidal changes after anti-VEGF therapy in neovascular-AMD patients: a systematic review and meta-analysis of SD-OCT studies. Surv Ophthalmol.

[6] Ferløv Baselius NJ, Brynskov T, Falk MK, Sørensen TL, Subhi Y (2021). Driving vision in patients with neovascular AMD in anti-VEGF treatment. Acta Ophthalmol.

[7] Subhi Y, Schneider M, Hajari JN, la Cour M (2024). Injection burden and treatment intervals of aflibercept in observe-and-plan regimen for neovascular age-related macular degeneration. Acta Ophthalmol.

[8] Pugazhendhi A, Hubbell M, Jairam P, Ambati B (2021). Neovascular macular degeneration: a review of etiology, risk factors, and recent advances in research and therapy. Int J Mol Sci.

[9] Penha FM, Masud M, Khanani ZA, Thomas M, Fong RD, Smith K, Chand A, Khan M, Gahn G, Melo GB, Khanani AM (2024). Review of real-world evidence of dual inhibition of VEGF-A and ANG-2 with faricimab in NAMD and DME. Int J Retina Vitreous.

[10] Heier JS, Singh RP, Wykoff CC, Csaky KG, Lai TYY, Loewenstein A, Schlottmann PG, Paris LP, Westenskow PD, Quezada-Ruiz C (2021). The angiopoietin/tie pathway in retinal vascular diseases: a review. Retina.

[11] Ferro Desideri L, Traverso CE, Nicolò M (2022). The emerging role of the angiopoietin-tie pathway as therapeutic target for treating retinal diseases. Expert Opin Ther Targets.

[12] Heier JS, Khanani AM, Quezada Ruiz C, Basu K, Ferrone PJ, Brittain C, Figueroa MS, Lin H, Holz FG, Patel V, Lai TYY, Silverman D, Regillo C, Swaminathan B, Viola F, Cheung CMG, Wong TY, TENAYA and LUCERNE Investigators (2022). Efficacy, durability, and safety of intravitreal faricimab up to every 16 weeks for neovascular age-related macular degeneration (TENAYA and LUCERNE): two randomised, double-masked, phase 3, non-inferiority trials. Lancet.

[13] Wykoff CC, Abreu F, Adamis AP, Basu K, Eichenbaum DA, Haskova Z, Lin H, Loewenstein A, Mohan S, Pearce IA, Sakamoto T, Schlottmann PG, Silverman D, Sun JK, Wells JA, Willis JR, Tadayoni R, YOSEMITE and RHINE Investigators (2022). Efficacy, durability, and safety of intravitreal faricimab with extended dosing up to every 16 weeks in patients with diabetic macular oedema (YOSEMITE and RHINE): two randomised, double-masked, phase 3 trials. Lancet.

[14] Chen D (2022). Real-world studies: bridging the gap between trial-assessed efficacy and routine care. J Biomed Res.

[15] Dang A (2023). Real-world evidence: a primer. Pharmaceut Med.

[16] Higgins J, Thomas J, Chandler J, Cumpston M, Li T, Page M, Welch V (editors). Cochrane handbook for systematic reviews of interventions version 6.4 (updated August 2023). Cochrane 2023. www.training.cochrane.org/handbook. Accessed 28 May 2023.

[17] Moher D, Liberati A, Tetzlaff J, Altman DG, PRISMA Group (2009). Preferred reporting items for systematic reviews and meta-analyses: the PRISMA statement. BMJ.

[18] Stang A (2010). Critical evaluation of the Newcastle-Ottawa scale for the assessment of the quality of nonrandomized studies in meta-analyses. Eur J Epidemiol.

[19] Cheng AM, Joshi S, Banoub RG, Saddemi J, Chalam KV (2023). Faricimab effectively resolves intraretinal fluid and preserves vision in refractory, recalcitrant, and nonresponsive neovascular age-related macular degeneration. Cureus.

[20] Grimaldi G, Cancian G, Rizzato A, Casanova A, Perruchoud-Ader K, Clerici M, Consigli A, Menghini M (2024). Intravitreal faricimab for neovascular age-related macular degeneration previously treated with traditional anti-VEGF compounds: a real-world prospective study. Graefes Arch Clin Exp Ophthalmol.

[21] Hara C, Suzue M, Fujimoto S, Fukushima Y, Sayanagi K, Nishida K, Maruyama K, Sato S, Nishida K (2024). Comparison of loading dose between aflibercept and faricimab for neovascular age-related macular degeneration. J Clin Med.

[22] Hikichi T (2023). Investigation of satisfaction with short-term outcomes after switching to faricimab to treat neovascular age-related macular degeneration. Jpn J Ophthalmol.

[23] Inoda S, Takahashi H, Takahashi R, Hashimoto Y, Yoshida H, Takahashi H, Takayama T, Kawashima H, Yanagi Y (2023). Visual and anatomical outcomes after initial intravitreal faricimab injection for neovascular age-related macular degeneration in patients with prior treatment history. Ophthalmol Ther.

[24] Kataoka K, Itagaki K, Hashiya N, Wakugawa S, Tanaka K, Nakayama M, Yamamoto A, Mukai R, Honjyo J, Maruko I, Kawai M, Miyara Y, Terao N, Wakatsuki Y, Onoe H, Mori R, Koizumi H, Sekiryu T, Iida T, Okada AA, Japan AMD Research Consortium (JARC) (2024). Six-month outcomes of switching from aflibercept to faricimab in refractory cases of neovascular age-related macular degeneration. Graefes Arch Clin Exp Ophthalmol.

[25] Khanani AM, Aziz AA, Khan H, Gupta A, Mojumder O, Saulebayeva A, Abbey AM, Almeida DRP, Avery RL, Banda HK, Barakat MR, Bhandari R, Chang EY, Haug SJ, London NJS, Mein L, Sheth VS, Wolfe JD, Singer MA, Danzig CJ (2023). The real-world efficacy and safety of faricimab in neovascular age-related macular degeneration: the TRUCKEE study—6 month results. Eye.

[26] Kishi M, Miki A, Kamimura A, Okuda M, Matsumiya W, Imai H, Kusuhara S, Nakamura M (2023). Short-term outcomes of faricimab treatment in aflibercept-refractory eyes with neovascular age-related macular degeneration. J Clin Med.

[27] Leung EH, Oh DJ, Alderson SE, Bracy J, McLeod M, Perez LI, Bottini A, Chin Yee D, Mukkamala K (2023). Initial real-world experience with faricimab in treatment-resistant neovascular age-related macular degeneration. Clin Ophthalmol.

[28] Maruyama-Inoue M, Yanagi Y, Inoue T, Kadonosono K (2024). Comparison of functional and morphologic changes between brolucizumab and faricimab in neovascular age-related macular degeneration. Graefes Arch Clin Exp Ophthalmol.

[29] Matsubara H, Nagashima R, Chujo S, Matsui Y, Kato K, Kuze M, Kondo M (2023). Subclinical ocular changes after intravitreal injections of different anti-VEGF agents for neovascular age-related macular degeneration. J Clin Med.

[30] Matsumoto H, Hoshino J, Nakamura K, Akiyama H (2024). One-year results of treat-and-extend regimen with intravitreal faricimab for treatment-naïve neovascular age-related macular degeneration. Jpn J Ophthalmol.

[31] Mukai R, Kataoka K, Tanaka K, Miyara Y, Maruko I, Nakayama M, Watanabe Y, Yamamoto A, Wakatsuki Y, Onoe H, Wakugawa S, Terao N, Hasegawa T, Hashiya N, Kawai M, Maruko R, Itagaki K, Honjo J, Okada AA, Mori R, Koizumi H, Iida T, Sekiryu T (2023). Three-month outcomes of faricimab loading therapy for wet age-related macular degeneration in Japan. Sci Rep.

[32] Ng B, Kolli H, Ajith Kumar N, Azzopardi M, Logeswaran A, Buensalido J, Mushtaq B, Chavan R, Chong YJ (2024). Real-world data on faricimab switching in treatment-refractory neovascular age-related macular degeneration. Life.

[33] Pandit SA, Momenaei B, Wakabayashi T, Mansour HA, Vemula S, Durrani AF, Pashaee B, Kazan AS, Ho AC, Klufas M, Regillo C, Yonekawa Y, Hsu J, Kuriyan A, Chiang A (2024). Clinical outcomes of faricimab in patients with previously treated neovascular age-related macular degeneration. Ophthalmol Retina.

[34] Raimondi R, Falfeli T, Bogdanova-Bennet A, Varma D, Habib M, Kotagiri A, Steel DH, Grinton M (2023). Outcomes of treatment-resistant neovascular age-related macular degeneration switched from aflibercept to faricimab. Ophthalmol Retina.

[35] Rush RB (2023). One-year outcomes of faricimab treatment for aflibercept-resistant neovascular age-related macular degeneration. Clin Ophthalmol.

[36] Schneider M, Bjerager J, Hodzic-Hadzibegovic D, Klefter ON, Subhi Y, Hajari J (2024). Short-term outcomes of treatment switch to faricimab in patients with aflibercept-resistant neovascular age-related macular degeneration. Graefes Arch Clin Exp Ophthalmol.

[37] Stanga PE, Valentín-Bravo FJ, Stanga SEF, Reinstein UI, Pastor-Idoate S, Downes SM (2023). Faricimab in neovascular AMD: first report of real-world outcomes in an independent retina clinic. Eye.

[38] Szigiato A, Mohan N, Talcott KE, Mammo DA, Babiuch AS, Kaiser PK, Ehlers JP, Rachitskaya A, Yuan A, Srivastava SK, Sharma S (2024). Short-term outcomes of faricimab in patients with neovascular age-related macular degeneration on prior anti-VEGF therapy. Ophthalmol Retina.

[39] Tamiya R, Hata M, Tanaka A, Tsuchikawa M, Ueda-Arakawa N, Tamura H, Miyata M, Takahashi A, Kido A, Muraoka Y, Miyake M, Ooto S, Tsujikawa A (2023). Therapeutic effects of faricimab on aflibercept-refractory age-related macular degeneration. Sci Rep.

[40] Tanaka A, Hata M, Tsuchikawa M, Ueda-Arakawa NU, Tamura H, Miyata M, Takahashi A, Kido A, Muraoka Y, Miyake M, Ooto S, Tsujikawa A (2024). Short-term outcomes of 3 monthly intravitreal faricimab on different subtypes of neovascular age-related macular degeneration. Clin Ophthalmol.

[41] Foxton RH, Uhles S, Grüner S, Revelant F, Ullmer C (2019). Efficacy of simultaneous VEGF-A/ANG-2 neutralization in suppressing spontaneous choroidal neovascularization. EMBO Mol Med.

[42] Tadayoni R, Sararols L, Weissgerber G, Verma R, Clemens A, Holz FG (2021). Brolucizumab: a newly developed anti-VEGF molecule for the treatment of neovascular age-related macular degeneration. Ophthalmologica.

[43] Baumal CR, Bodaghi B, Singer M, Tanzer DJ, Seres A, Joshi MR, Feltgen N, Gale R (2021). Expert opinion on management of intraocular inflammation, retinal vasculitis, and vascular occlusion after brolucizumab treatment. Ophthalmol Retina.

[44] Prenner JL, Halperin LS, Rycroft C, Hogue S, Williams Liu Z, Seibert R (2015). Disease burden in the treatment of age-related macular degeneration: findings from a time-and-motion study. Am J Ophthalmol.

