# Supplementary material for: Real-world efficacy of intravitreal faricimab for neovascular age-related macular degeneration: a systematic review
Source: Int J Retina Vitreous. 2024 Jul 12;10:48. doi: 10.1186/s40942-024-00566-0 (PMC11245828; doi:10.1186/s40942-024-00566-0)
Supplement: Supplementary file 1 — Additional file 1. Details of the literature search across different databases. [file 40942_2024_566_MOESM1_ESM.docx]

**Supplementary file 1.** Details of the literature search across different databases.

**PubMed:**


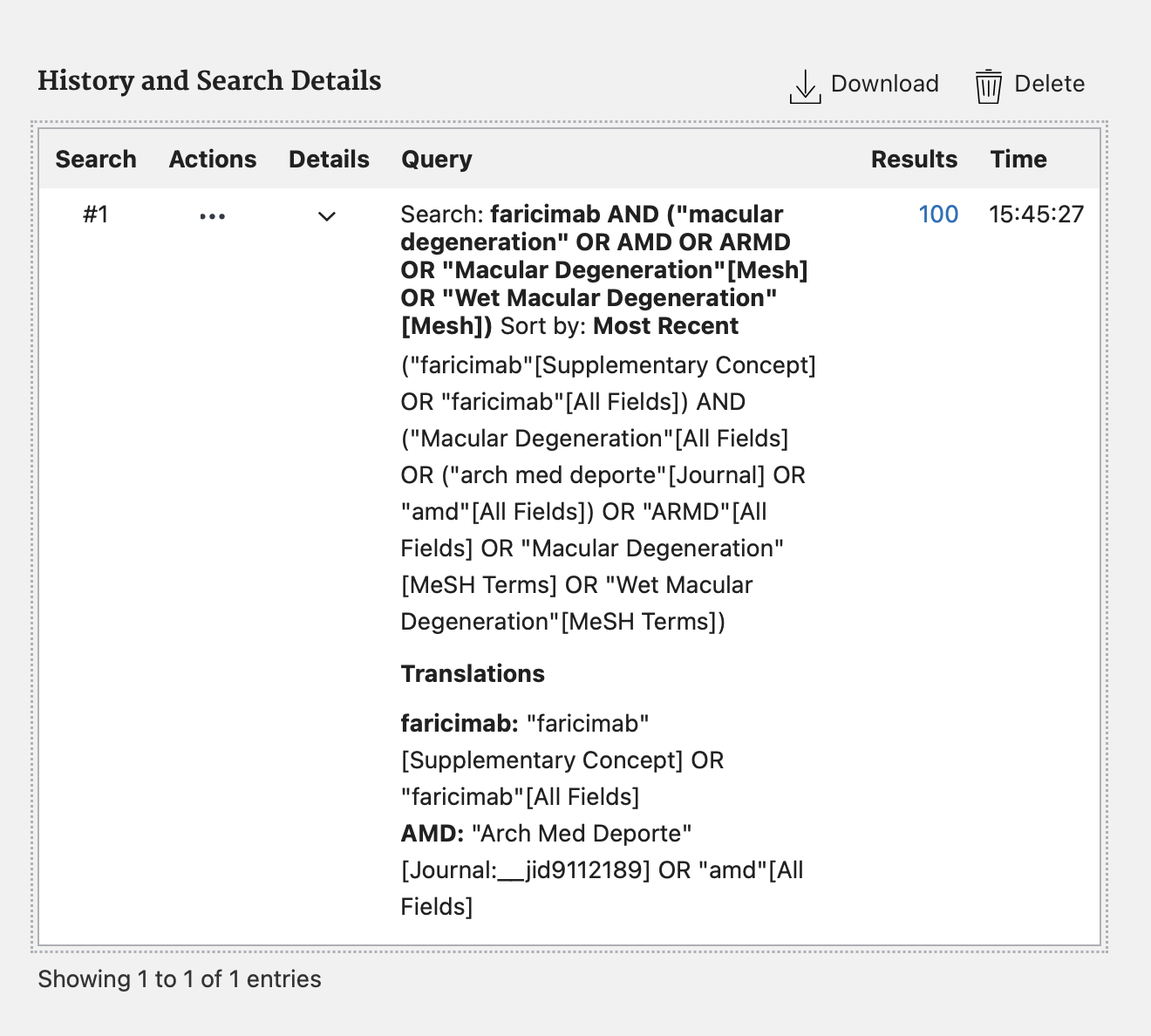


**Embase:**


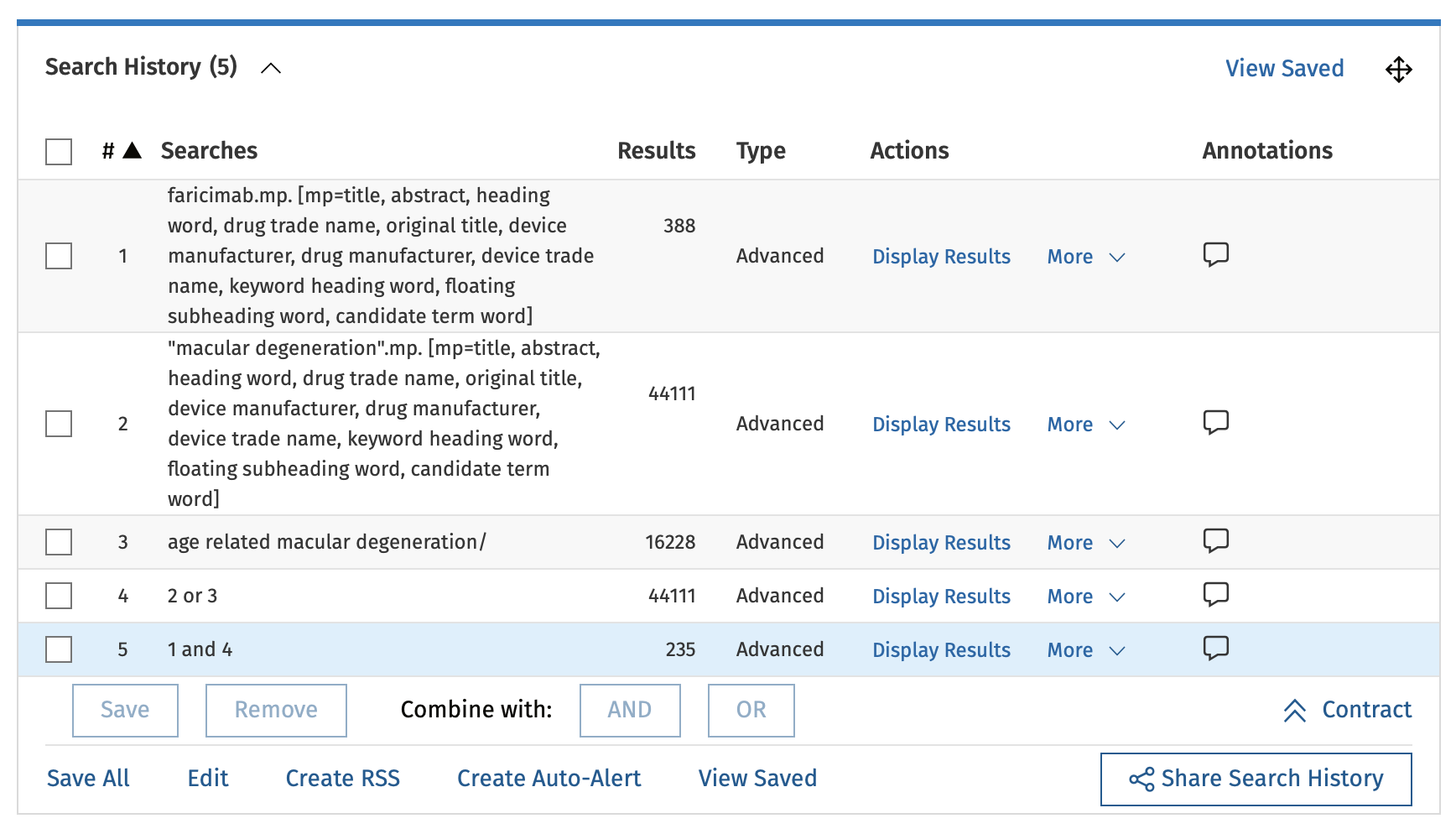


**Web of Science Core Collection, BIOSIS Previews, Current Contents Connect, Data Citation Index, Derwent Innovations Index, KCI-Korean Journal Database, ProQuest ™ Dissertations & Theses Citation Index, SciELO Citation Index:**


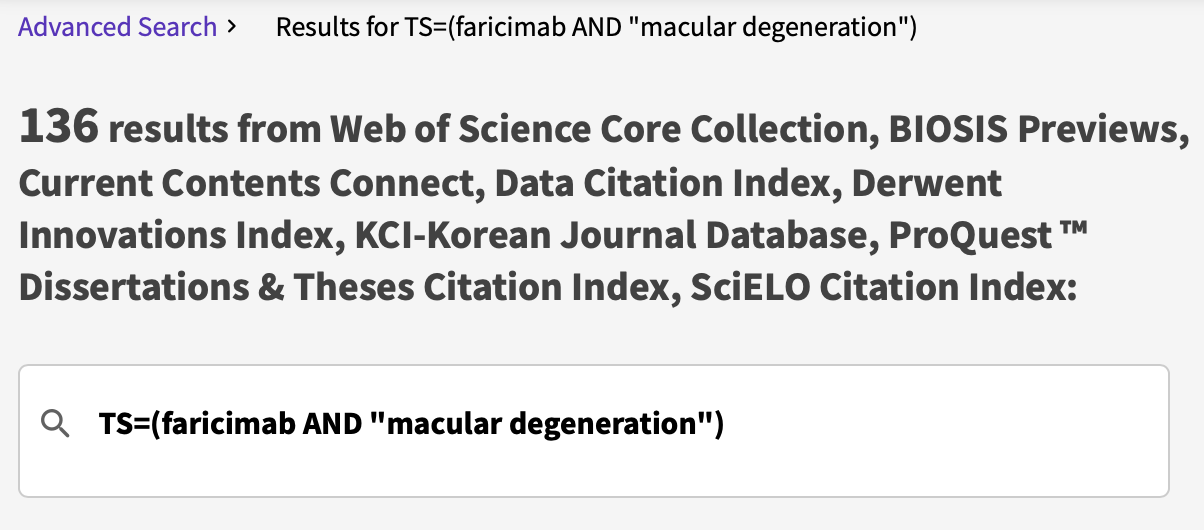


**Cochrane Library:**

Search Name:

Date Run: 17/03/2024 16:53:13

Comment:

ID Search Hits

#1 faricimab 85

#2 "macular degeneration" 3988

#3 #1 AND #2 38
